# Clinical outcomes of chemotherapy in cancer patients with different ethnicities

**DOI:** 10.1002/cnr2.1830

**Published:** 2023-05-07

**Authors:** Suhrud Pathak, Kelsee K. Zajac, Manjusha Annaji, Manoj Govindarajulu, Rishi M. Nadar, Dylan Bowen, R. Jayachandra Babu, Muralikrishnan Dhanasekaran

**Affiliations:** ^1^ Department of Drug Discovery and Development, Harrison College of Pharmacy Auburn University Auburn Alabama USA; ^2^ Department of Pharmacology and Experimental Therapeutics, College of Pharmacy and Pharmaceutical Sciences University of Toledo Toledo Ohio USA

**Keywords:** cancer, chemotherapeutics, dosage forms, ethnic variation, racial variation, treatment outcomes

## Abstract

**Background:**

Choosing the most effective chemotherapeutic agent with safest side effect profile is a common challenge in cancer treatment. Although there are standardized chemotherapy protocols in place, protocol changes made after extensive clinical trials demonstrate significant improvement in the efficacy and tolerability of certain drugs. The pharmacokinetics, pharmacodynamics, and tolerance of anti‐cancer medications are all highly individualized. A driving force behind these differences lies within a person's genetic makeup.

**Recent findings:**

Pharmacogenomics, the study of how an individual's genes impact the processing and action of a drug, can optimize drug responsiveness and reduce toxicities by creating a customized medication regimen. However, these differences are rarely considered in the initial determination of standardized chemotherapeutic protocols and treatment algorithms. Because pharmacoethnicity is influenced by both genetic and nongenetic variables, clinical data highlighting disparities in the frequency of polymorphisms between different ethnicities is steadily growing.  Recent data suggests that ethnic variations in the expression of allelic variants may result in different pharmacokinetic properties of the anti‐cancer medication. In this article, the clinical outcomes of various chemotherapy classes in patients of different ethnicities were reviewed.

**Conclusion:**

Genetic and nongenetic variables contribute to the interindividual variability in response to chemotherapeutic drugs. Considering pharmacoethnicity in the initial determination of standard chemotherapeutic protocols and treatment algorithms can lead to better clinical outcomes of patients of different ethnicities.

## 1 INTRODUCTION

The unregulated growth of poorly differentiated cells with the ability to invade surrounding and/or distant tissues is a defining feature of cancer. At times, these malignant cells can spread throughout the body, which severely disrupts organ function, leading to significant morbidity and mortality.^1^ According to the International Agency for Research on Cancer (IARC), there were more than 17 million new cases of cancer worldwide and approximately 10 million cancer‐related mortalities in the year 2018. It is projected that in 2040 there will be 28 million new cancer cases and 16 million cancer‐related deaths.^2^ This projection highlights the potential drastic escalation of the global cancer burden, which is likely due to the increase in worldwide prevalence of several inciting risk factors. An increase in the aging population, smoking habits, unhealthy diet, and sedentary lifestyle all contribute to the growing number of new malignancies which tend to display a more aggressive presentation with an higher mortality rate. Other exacerbating factors include iatrogenesis and the lack of proper pharmacotherapeutics in developing nations. Treating cancer varies by origin and can be achieved through different modalities such as: chemotherapy, radiation, surgical removal, immunotherapy, hormonal therapy, or stem cell/bone marrow transplant.^3^ Outside of primary therapy, additional treatments may be divided into specific categories depending on their rationale and timing. Neoadjuvant therapy is delivered to reduce the size of the tumor and increase the cells' susceptibility to the primary treatment prior to its administration^4^ Adjuvant therapy prevents the reemergence of malignant cells that remain past initial treatment. Lastly, palliative treatment is not seen as curative, however, it aims to manage the symptoms of cancer as well as any potential side effects of cancer treatment.^5^ The major chemotherapeutic classes available are alkylating agents, nitrosoureas, antimetabolites, antitumor antibiotics, plant alkaloids, biological response modifiers, and hormonal anti‐cancer agents. Despite the evolution of novel therapeutic approaches and an increased understanding of the pathophysiology behind certain cancers, there are discrepancies in the clinical outcomes depending on an individual's racial background or ethnicity after chemotherapy treatment.

With chemotherapy administration, the primary goal is to identify the dose of drug with the greatest efficacy while minimizing adverse effects. These cytotoxic agents undergo rigorous testing through randomized clinical trials to determine the optimal dose that takes this into consideration. A standardized dosing scheme is then recommended from the finalized clinical trial. Routinely, further dose adjustments may vary per individual based on body surface area or renal clearance. Several interindividual differences play a critical role in the variation of pharmacological efficacy, adverse effects, hypersensitivity reactions, and drug interactions despite receiving similar chemotherapeutic doses. One factor with the ability to affect drug disposition is an individual's ethnic background. Variations in pharmacokinetic and pharmacodynamic properties have been observed between ethnic populations which can influence therapeutic results.^6^ The term pharmacoethnicity involves the study of nongenetic and genetic factors that affect the differences in drug response or toxicity based on an individual's ethnicity. The majority of drugs target enzymes, receptors, ion‐channels, pumps, transporters, and nucleic acids (DNA/RNA).^7^ Within the last decade, advancements in pharmacogenomics have identified genetic polymorphisms in the expression and/or activities of human drug targets specific to an individual's ethnicity.^8^ This change is also seen within enzymes responsible for the metabolism of certain chemotherapeutics. These polymorphisms may influence drug disposition in certain populations. Any deviation in the pharmacokinetic and pharmacodynamic properties can lead to adverse outcomes when dosing is decided without the consideration of these changes. Alterations in pharmacokinetics may be caused by variations in allele frequencies and types of allelic variants of nuclear receptors, transporters, and drug‐metabolizing enzymes in individuals from different ethnic backgrounds.^9^, ^10^ Several variations in drug metabolizing enzymes and transporter proteins have been identified. Additionally, changes in protein structure can either cause the protein to decrease or increase its function ultimately changing the PK and PD parameters of certain drugs. These alterations can also lead to chemotherapeutic resistance.^11^ Genetic pharmacoethnicity could be a critical contributing factor to the disparities seen in chemotherapeutic clinical outcomes of individuals of different backgrounds.

Outside of genetic polymorphisms, several other nongenetic factors can influence the clinical outcomes of cancer patients of different ethnicities. These factors include the lack of access to healthcare, suboptimal use of therapy, and lower utilization of cancer screening methods. All of these discrepancies can lead to a more aggressive tumor biology and stage at presentation which contributes to the poorer outcomes observed in some ethnic minorities.^12^ A patient's socioeconomic status, education, insurance, misconceptions, and preferences can all impact the care received. One study revealed that African American Medicare beneficiaries were less likely to encounter specialists and high‐quality imaging compared to their Caucasian counterparts.^13^ Additionally, African American patients living further away from cancer treatment centers or areas of higher poverty underwent less radiation after breast‐conserving therapy.^14^ A detailed study of cancer risk factors based on ethnicity revealed that people of Hispanic, Asian, and American Indian/Alaska Native descent are less likely to undergo screening for breast, cervical, and colorectal cancers compared to their Caucasian and African American counterparts.^15^ Additionally, patient misconceptions and preferences also play a role in the disparity of cancer treatment outcomes. In one study, African American patients felt that their quality of life would worsen after lung cancer surgery which resulted in fewer surgical resections compared to Caucasian patients.^16^ These factors all contribute heavily to the disparities observed in clinical outcomes seen with ethnic minorities and cannot be ignored when devising solutions to address them.

Part of the cancer disparities observed between ethnicities may be attributed to the increased frequency of specific germ line and somatic mutations known to cause certain types of cancer. Most cancer types are associated with a high‐mutation burden^17^ either from an increased susceptibility to genetic mutations or due to mutagenic environmental exposure. For example, African American males have the highest rate of aggressive prostate cancer mortality compared to other ethnicities. A possible reason for this finding is due to differences in the frequency of specific tumor mutations within prostate cancer of African American men. In one study, African American males displayed less somatic alterations of Transmembrane serine protease 2‐ erythroblast transformation‐specific‐related gene (TMPRSS2‐ERG), less Phosphatase and tensin homolog (PTEN) deletions, and higher overexpression of serine peptidase inhibitor Kazal type 1 (SPINK1) compared to Caucasian males. SPINK1 mutation is associated with a more aggressive phenotype of prostate cancer.^18^ Furthermore, in the case of lung cancer, activating epidermal growth factor receptor (EGFR) mutations (base pair deletion in exon 19 and point mutation in exon 21) are significantly higher in people of Chinese, Japanese, and Korean descent compared to Caucasian individuals in Europe and the United States.^19^ Findings of genetic variations of cancer inducing mutations between different populations highlights an important factor to consider for treatment choice and outcomes. Therefore, expanding the awareness of inherited and somatic mutations across populations can be used to improve the strategies for cancer prevention and treatment.^11^ Different clinical biomarkers are also implicated among different ethnic populations as listed in Table 1. Population‐based differences have shown to impact chemotherapeutic treatment and safety outcomes.^20^ Several studies have proven variations in chemotherapy drug responsiveness among different ethnicities as well as differences in clinical outcomes.^10^, ^19^ This review will focus on the treatment outcomes based on different types of chemotherapeutic agents in patients of different ethnicities.

**TABLE 1:** Prevalence of clinical biomarkers and their implications across different ethnic groups^33^

Cancer	Biomarker	Prevalence	Clinical implication	References
Colorectal cancer	KRAS	Non‐Hispanic white: 15%; African American: 23%	Predicts the lack of response to EGFR antibodies	^34^
	MSI	Non‐Hispanic white: 9%; African American: 9%	Predicts the response to immune checkpoint inhibitors	^34^
Breast cancer	HER2 amplification	White: 13%; Asian: 20%; Black:17%	Predict the sensitivity to HER2 targeting antibodies	^35^
	Triple negative i.e. lacks, PR, ER and HER2	Caucasian (United Kingdom): 14.5%; African Descent (Nigeria): 48.1%	Predicts the sensitivity to PARP inhibitors and platinum chemotherapy	^36^
Prostate cancer	PTEN	Caucasian (United Kingdom): 29.7%; White (Others):19.8%; Chinese: 5.4%; African American: 6.9%	Predicts the sensitivity to PI3K inhibitors	^18^, ^37^
	TMPRSS‐ERG	White Americans: 50%; Japanese: 15.9%; African Americans: 31.3%	Predict the sensitivity to abiraterone acetate	^38^
	PSA	Higher baseline PSA levels and faster PSA doubling time in Black men than White men	Black men may benefit from earlier and more aggressive screening and treatment	^39^, ^40^
Gastric cancer	HER2 amplification	Europe: 23.6%; Central/South America: 16.1%; Asia pacific: 23.9%	Predicts the sensitivity to HER2 targeting antibodies	^41^
Melanoma	BRAF	Higher prevalence of BRAF mutations in White populations than other racial groups	BRAF‐targeted therapy (vemurafenib, dabrafenib) can be effective in patients with BRAF‐mutant tumors	^42^, ^43^
Ovarian Cancer	CA‐125	CA‐125 levels are generally lower in Asian women than Western women, making it a less useful screening tool in Asian populations	CA‐125 can be used for monitoring disease progression and response to treatment	^44^, ^45^
Lung cancer	ALK	White: 5.6%; Chinese: 6.8%	Predicts the sensitivity to ALK inhibitors	^46^, ^47^
	KRAS	White: 31.6%; Chinese: 10.9%	Predicts resistance to EGFR tyrosine kinase inhibitors	^48^
	EGFR	White: 6%; White (United States: 14%; Australia:7%; Japan: 27%; Taiwan: 34%; African American: 8.7%	Predicts the sensitivity to EGFR tyrosine kinase inhibitors such as erlotinib, gefitinib	^19^, ^49^

Abbreviations: ALK, anaplastic lymphoma kinase; BRAF, v‐raf murine sarcoma viral oncogene homolog B1; CA‐125, cancer antigen 125; EGFR, epidermal growth factor receptor; ERG, erythroblast‐transformation‐specific (ETS) Related Gene; HER2, human epidermal growth factor receptor 2; KRAS, Kirsten rat sarcoma viral oncogene; MSI, microsatellite instability; PI3K, phosphoinositide 3‐kinases; PRAP, poly ADP ribose polymerase; PSA, Phosphoinositide 3‐kinases; PSA, Prostate‐specific antigen; PTEN, Phosphatase and tensin homolog gene; TMPRSS, transmembrane serine protease 2.

## 2 PREVALENCE OF CANCER IN MINORITY ETHNIC POPULATIONS

### 2.1 American Indian/Alaska Native

A primary reason for loss of life in American Indian or Alaska Native individuals is cancer related. American Indian/Alaska Natives have a higher risk of developing lung, colorectal, liver, stomach, and kidney cancer and a lower rate of breast cancer as compared to non‐Hispanic Caucasian patients. More specifically, American Indian/Alaska Native women are 20% more likely to develop renal cancer compared to non‐Hispanic Caucasian women. American Indian/Alaska Native men are 30% more likely to develop stomach cancer compared to non‐Hispanic Caucasian men.^21^ Additionally, American Indian and Alaska Natives display the highest incidence of colorectal cancer compared to other ethnic populations (ACS citation). This increased threat of colorectal cancer in American Indian/Alaska Natives is thought to be attributed to differences observed in diet, lifestyle, and genetic makeup.

### 2.2 Asian

One of the leading causes of death among Asian populations is cancer.^22^ Asian Americans display lower rates of cancer overall compared to non‐Hispanic Caucasian Americans; however, disparities exist among the specific cancer types.^23^ In 2020, the most common incidences of cancer among Asian populations were lung, breast, and colorectal cancer with lung cancer being the leading cause of cancer‐related mortality. More specific to Korea, Japan, and Kuwait, the incidence of cancer is rising among young females. This is thought to be due to increased screening programs, changes in reproductive factors, and a delayed tobacco epidemic in women^24^, ^25^ (Harris, 1983 #2210). Overall, cancer‐related mortality is declining within Asian countries.^26^

### 2.3 Black or African American

Of all ethnic minority populations, Black or African American individuals have the highest rates of cancer‐related mortality for most cancer types. Common cancers observed within this population originate from the prostate, breast, lung, and colon/rectum.^27^ African American men were 1.2 and 1.7 times more likely to develop colon or prostate cancer from 2015 to 2019 compared to non‐Hispanic Caucasian men. In the same time frame, the incidence of breast cancer was approximately the same between African American and Caucasian women, however, African American women were 40 percent more likely to die from breast cancer. Similarly, African American men were twice as likely to succumb from prostate cancer when compared to non‐Hispanic Caucasian men.^28^ Despite this evidence, Black or African American individuals are severely underrepresented in clinical cancer trials.^22^

### 2.4 Hispanic or Latino

Hispanic individuals generally have lower cancer occurrence and mortality rates compared to non‐Hispanic Caucasian groups; however, certain disparities exist depending on cancer type. Hispanic individuals are seen to have higher rates of infection‐related cancers such as stomach or liver cancers.^29^ Hispanic women are at higher risk of developing and dying from cervical cancer compared to non‐Hispanic Caucasian women. The same findings are observed in stomach or liver cancer for both Hispanic men and women.^30^

### 2.5 Native Hawaiian or other Pacific Islander

One of the leading causes of death among Native Hawaiian or other Pacific Islander people is cancer.^22^ A significant cancer disparity seen among this group is in breast cancer incidence and mortality. One study of 6221 women found that Native Hawaiian or other Pacific Islander women were at a higher risk of developing secondary invasive breast cancer when compared to non‐Hispanic Caucasian women.^31^ Similarly, cervical cancer is twice as likely to be diagnosed in American Samoan women compared to non‐Hispanic Caucasian women. Liver cancer is more likely to develop in American Samoan and Native Hawaiian men when compared to non‐Hispanic Caucasian men. Between the years 2013–2015, the cancer mortality rate was highest for all cancer types in Native Hawaiians compared to non‐Hispanic Caucasians within the state.^32^

**FIGURE 1:**
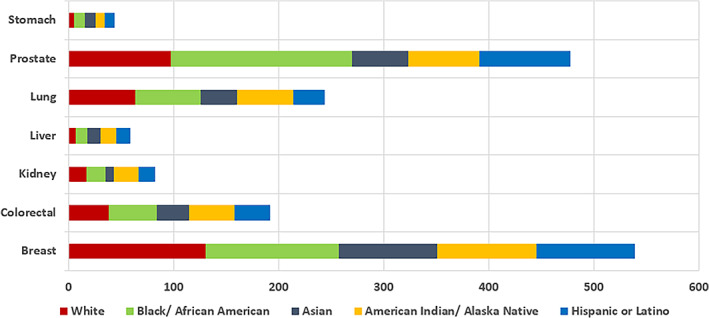
Incidence rates for different cancers by race and ethnicity, 2014–2018.^159^

**FIGURE 2:**
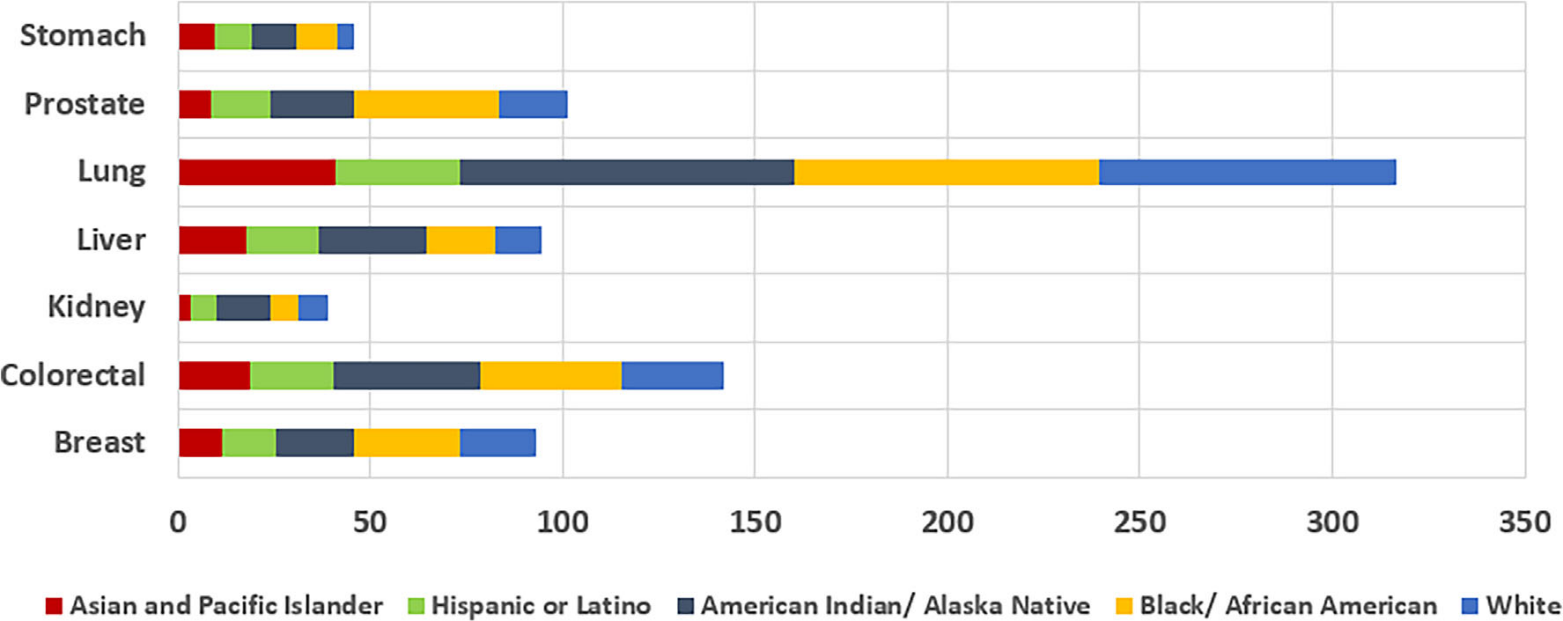
Death rates for different cancers by race and ethnicity, 2016–2020. Average annual rate per 100 000 population.^160^

Cancer prevalence based on ethnicity is summarized in the paragraphs above and the specific prevalence of certain clinical biomarkers specific to ethnicity are listed in Table 1. The incidence rates and death rates for different cancers by race and ethnicity from 2014 to 2018 is displayed in Figure 1 and Figure 2, respectively.

Based on the data presented, there is an imminent need to explore the outcomes influenced by differences in chemotherapeutic dosage forms, routes of administration, pharmacokinetics, pharmacodynamics, and toxic effects in patients of various ethnic backgrounds. This current study focuses on the disparities in the efficacy of commonly used chemotherapeutic drug classes such as, alkylating agents, antimetabolites, antitumor antibiotics, and other miscellaneous chemotherapeutics.

## 3 LITERATURE SEARCH METHODS

This study was performed by manually searching published scientific research articles through March 2023 from the following databases: PubMed central, Medline, Google Books, Science.gov, Worldwide Science, and Google Scholar. Search terms included the following: “Chemotherapy”, “Chemotherapeutics”, “Ethnicity”, “Racial”, and “Therapeutic outcome.” Relevant clinical content was obtained using CDC and Lexi‐Comp. References of selected articles were manually searched for additional information. A total of 159 articles were covered in our study. We encompassed clinical trials, journal articles, meta‐analyses, randomized control trials, reviews, and systematic reviews. Articles were searched under English language restrictions.

## 4 RESULTS

The definition of ethnicity and ethnic groups are variable based on country, region, background, and individual basis. For this reason and the specific studies investigated, this review will focus mainly on the five‐race classifications and will include pertinent information regarding specific ethnic groups where possible.

## 5 ALKYLATING AGENTS

The first nonhormonal medications to successfully treat cancer were alkylating agents.^50^ Alkylating agents exert their anti‐cancer activities primary through DNA alkylation, a process in which alkyl groups are added to the DNA bases. This induces DNA strand breaks and DNA crosslinking which leads to cell death.^51^ Alkylation also results in the production of different DNA adducts, which in turn prevent the proliferation of tumor cells. In aqueous solutions under physiological conditions, alkylating agents are a diversified class of chemical substances that share the property of generating positively charged (electrophilic, electron‐deficient) alkyl groups. The negatively charged (nucleophilic, electron‐rich) groups found in DNA and proteins/peptides can react with the positively charged alkyl groups. These interactions add alkyl groups to oxygen, nitrogen, phosphorous, or sulfur atoms (nucleophilic centers) which disrupt the biological activity of DNA and proteins. In terms of their anti‐cancer efficacy, alkylating drugs' interactions with DNA nucleobases are the most significant.^52^ Alkylating agents are administered majorly via the intravenous route while some of them are given orally.

Cyclophosphamide, an alkylating agent, is converted to its active form via the cytochrome P450 (CYP) enzymes CYP3A4, CYP2B6, and CYP2C9.^53^, ^54^ The role of CYP enzymes in anti‐cancer pharmacokinetics has been extensively discussed, and it is widely known that CYP enzymes behave differently depending on specific ethnic groups. For instance, in breast cancer patients, CYP3A4, CYP3A5, and CYP2B6 polymorphisms were more prevalent in African Americans compared with Caucasians. This led to the hypothesis that certain polymorphisms that prevent the activation of cyclophosphamide could result in ethnic‐specific drug exposure differences.^55^ This hypothesis was further confirmed in a lupus nephritis study wherein 95% of Caucasian patients treated with cyclophosphamide retained renal function on 5‐years follow‐up, compared with only 58% of African Americans.^56^ Similarly, a study by Isenberg et al. showed that African Americans and Hispanic patients treated for lupus nephritis showed lower response to cyclophosphamide treatment.^57^ Despite this, it has been hypothesized that the ethnic disparities in CYP3A polymorphism rates between Caucasians, Asians, American Indians, and African Americans may account for variations in chemotherapy outcomes.^11^

Platinum‐based drugs (cisplatin, carboplatin, and oxaliplatin) are another form of alkylating agents which are widely used for the treatment of many types of cancer.^58^ Several studies noted an increased frequency of toxicities (mainly hematological) in Asian patients in comparison to non‐Asians (Caucasians) treated with standard doses of platinum‐based drugs.^59^, ^60^ Interestingly, the incidence of these toxicities was still higher among Asians despite appropriate dose reduction.^11^ In a study comparing Russian (Caucasians) and Yakut (North Asians) ovarian cancer patients receiving cisplatin‐based chemotherapy, Single‐nucleotide polymorphism (SNPs) in more than 30 genes were found to be associated with several clinical end points such as tumor response, progression‐free survival, overall survival, and side effects. Some of the most important SNPs included glutathione S‐transferase (GST) genes (*GSTA1, GSTM1, GSTM3, GSTP1 and GSTT1*), DNA repair genes (*ERCC1, ERCC2 and XRCC1*) as well as *TP53* and *CYP2E1* genes. Indicating that the identified effects were specific for each of the two ethnic populations studies. For instance, Yakut patients showed increased frequency of nephrotoxicity and severe emesis with cisplatin in comparison to their Russian counterparts.^61^ Compared to other ethnicities, African Americans have a twice greater prevalence of multiple myeloma (MM). Both African American and non‐African American patients underwent autologous stem cell transplantation 24 h after receiving melphalan 200 mg/m^2^ intravenously in equally split dosages over two consecutive days. After autologous stem cell transplantation, patients from both African American and non‐African American backgrounds experienced equivalent treatment‐free survival, although overall survival was enhanced. In African American patients, post‐relapse survival increased, which indicated a greater response to salvage treatment. Based on these findings, there is a possibility of increased melphalan efficacy in African Americans when used in the treatment of multiple myeloma as compared to non‐African Americans.^62^ A study on the treatment of glioblastoma with Temozolomide showed a potential increase in efficacy by observing lower mortality rates of Asian/Pacific Islanders and Hispanic Caucasians compared to non‐Hispanic Caucasians. Another study demonstrated that genetic polymorphism (Leu84Phe) in the O^6^‐methylguanine‐DNA methyltransferase gene may increase the chance of Temozolomide resistance and confers a poor prognosis in individuals with malignant gliomas.^63^, ^64^

These findings indicate the existence of ethnicity‐related molecular mechanisms underlying the sensitivity of patients treated with platinum‐based drugs. These findings are listed in Table 2.

**TABLE 2:** Pharmacokinetics and Pharmacodynamics and racial/ethnic differences in the treatment outcomes with Alkylating agents. PK/PD data of various drugs was obtained from ref. [65, 66, 67, 68]

Drug	Route, dosage form and dose (human)	Pharmacokinetics (PK)	Pharmacodynamics (PD)	Ethnic variability of PK/PD
Altretamine	Oral Capsule; 260 mg/m^2^/day	A: Well‐absorbed D: Highly concentrated in the liver, kidney, & small intestine Tmax: 0.5–3 h t^1/2^: 7 h M: Liver; extensive demethylation E: Urine (90%; <1% as unchanged drug)	Covalent bonds formed between reactive intermediates with microsomal proteins and DNA potentially result in DNA damage. Metabolism is essential for cytotoxicity.	None reported
Bendamustine	Intravenous Ready‐to‐dilute solution‐100 mg/4 mL (25 mg/mL) Lyophilized powder for reconstitution‐ 25–100 mg/single‐dose vial	Protein Bound: 94%–96% Vd: 25 Liters t^1/2^: 40 min M: CYP1A2 (low amount) E: Feces (90%); urine (1–10%)	A non‐specific alkylating agent that cleaves the DNA by cross‐linking single or double DNA strands	A study of Japanese people found that bendamustine exposure was 40% greater than that of non‐Japanese subjects with lymphocytic leukemia. The observed difference was attributed to the differential hepatic cytochrome P450 1A2 dependent metabolism.^69^
Busulfan	Intravenous Injection; 0.8 mg/kg	Tmax serum: 5 min (IV) F: 68%–80% depending on age D: Protein bound: 32% Vd: 0.6–1.0 L/kg (adults); 1.4–1.6 L/kg (children) M: Liver Elimination t^1/2^: 2.5 h CL: 2.52 mL/min/kg E: Urine (25–60%)	Alkylating agent; inhibits DNA replication and RNA transcription; linkages DNA strands; has limited immunosuppression effect; impacts myeloid cells more than lymphoid cells; is extremely toxic to hematopoietic stem cells.	Glutathione‐transferase (GST) gene family (A1, P1, M1, and T1) is involved in busulfan metabolism. These genes may cause variations in busulfan concentrations. Genotyping (GSTA1, GSTP1, and GSTM1) in children undergoing hematopoietic stem cell transplantation might allow better prediction of busulfan metabolism and prevent dose related drug toxicity..^70^ No ethnicity/racial differences in PK/PD were found.
Carboplatin	Intravenous Injection; 10 mg/mL	Tmax: 2–4 h Protein bound: 87% (platinum) Vd: 16 L CL: 4.4 L/h E: Urine (70% as carboplatin) t^1/2^: Carboplatin: 3–6 h Total plasma platinum: 4–6 days	Platinum coordination molecule that bonds covalently to DNA and cross‐linkages DNA strands. Not a real alkylating agent.	A majority of Asian individuals received lower doses of carboplatin in lung cancer than Caucasians, their tumor response rate was more than twice as high.^71^
Carmustine	Intravenous Injection; 150–200 mg/m^2^	Elimination t^1/2^: 1.4 min (initial); 22 min (secondary) Vd: 3.25 L/kg D: Readily crosses blood–brain barrier CL: 56 mL/min/kg E: Urine (60–70%)	Alkylates and cross‐linking DNA and RNA, disrupting with regular DNA activity; carbamylation of aminoacids in protein may also disrupt enzyme activities.	None reported
Chlorambucil	Oral Tablets; 0.1 mg/kg	A: 100% F: 70%–80% Tmax: 1 hr Protein Bound: 99% Vd: 0.3 L/kg M: Liver Elimination t^1/2^: 1.5 h CL: 492 ± 160 ng/mL E: Urine (20–60% metabolites) Dialyzable: No	Alkylating substances from the nitrogen mustard family that cross‐linking DNA and disrupts with replicating DNA and RNA translation; agnostic to the cell cycle	None reported
Cisplatin	Intravenous Injection; 1 mg/mL	Elimination t^1/2^: 24 h to 47 days Protein bound: >90% E: Urine (90%); feces (10%) CL: 15 L/hr/m^2^ Vd: 11 L/m^2^	Platinum coordination compound that inhibits DNA synthesis; cross‐links and denatures strands of DNA; disrupts DNA function by covalently binding to DNA bases; can also produce DNA intrastrand cross‐linking and breakage	Yakut individuals with the Glutathione S‐transferase theta‐1 (GSTT1)‐null genotype were more likely to have nephrotoxicity in cisplatin‐based chemotherapy in ovarian cancer. Similarly, individuals with heterozygous excision repair cross complementation group 1 (ERCC1) genotypes in the Russian group had frequent nephrotoxicity.^72^
Cyclo‐phosphamide	Intravenous Injection; 40–50 mg/kg	F: 75% Tmax: 1 h; Protein bound: low metabolites, >60% Vd: 0.48–0.71 L/kg M: liver Elimination t^1/2^: 3–12 hr E: Urine	Metabolites interfere with malignant cell growth by cross‐linking tumor cell DNA; drug does not have specificity for any phase of the cell cycle; also has potent immunosuppressive activity	There is a possible decreased efficacy in African Americans compared with Caucasians in breast cancer.^55^ CYP3A4 also inactivates cyclophosphamide via conversion to dechloroethylcyclophosphamide, and breast cancer patients showed that many *CYP3A4* variant polymorphisms are more prevalent in African Americans compared with Caucasians.^55^, ^73^ Caucasian patients with lupus nephritis who were treated with cyclophosphamide retained renal function compared to African Americans.^56^
Dacarbazine	Intravenous Injection; 2–4.5 mg/kg	t^1/2^: 5 h (terminal) Cmax: 8 mcg/mL (4.5 mg/kg dose) Protein Bound: ~5% Vd: 0.6 L/kg M: Liver E: Urine (~40%)	Non‐cell cycle specific; alkylates DNA & RNA; results in DNA double strand breakage and apoptosis	None reported
Ifosfamide	Intravenous Injection; 1.2 g/m^2^/day	F: 90%–100% Tmax: 20–30 minutes Vd: 33 L M: liver Elimination t^1/2^: 15 h (high dose of 3800–5000 mg/m^2^); 7 hr (low dose of 1800–2400 mg/m^2^) E: Urine 70–86% (high dose of 5000 mg/m^2^); 12–16% (low dose of 1600–2400)	Synthetic analog of cyclophosphamide; cross‐links DNA strands; inhibits DNA synthesis and protein synthesis	None reported
Lomustine	Oral Capsules; 130 mg/m^2^	Elimination t^1/2^ (biphasic): 16–24 h (parent drug); 16–48 h (active metabolite) Cmax: ~3 h M: Liver Duration: 5–6 weeks (bone marrow recovery) E: Urine (50%); feces (<5%)	Unknown; DNA and RNA synthesis suppression triggered on by carbamylation of DNA polymerase, alkylation of DNA, and modification of RNA proteins.	None reported
Mechlo‐rethamine	Intravenous Injection; 0.4 mg/kg	Elimination t^1/2^: <1 min M: Extensive Excretion: Urine	Crosses DNA strands (interstrand and intrastrand crosslinking), which results in miscoding breakage and eventually terminates DNA replication. That hinders DNA and RNA synthesis by forming carbonium ions.	None reported

Abbreviations: A, absorption; CL, clearance; Cmax, peak plasma or serum concentration; D, distribution; E, excretion; F, bioavailability; M, metabolism; t½, half‐life; Tmax, time to maximum concentration; Vd, volume of distribution.

## 6 ANTIMETABOLITE CHEMOTHERAPY

Approximately 70 years ago, Farber discovered the potential anti‐cancer effect of aminopterin, an antimetabolite, by inducing remission in patients with acute leukemia. The antimetabolites are a class of anti‐cancer agents that mimic natural substrates structurally, however, their structure differs enough to obstruct their metabolism.^74^ Antimetabolites' primary mechanism of action is to cause depletion of nucleotides, which in turn halts DNA replication. Some antimetabolites are able to mimic nucleic acids falsely, creating structural defects that result in cell death via other pathways, such as DNA breakage.^75^ Common routes of administration for antimetabolites are orally, intravenously, and subcutaneously.

Notably, the prevalence of any complication with antimetabolite chemotherapy was lower in African Americans when compared to Caucasians. Reduced rates of complications included diarrhea, nausea and vomiting, and mucositis. The observation of skin toxicity demonstrated no difference based on ethnicity with the antimetabolite drugs. Germline polymorphisms in critical genes involved in antimetabolite toxicity and response may be responsible for the interethnic variations observed.^11^

Of the available antimetabolites, clinical differences are observed based on ethnicity in 5‐fluorouracil (5‐FU). 5‐fluorouracil is most commonly used in the setting of advanced stage colon cancer. Significant ethnic disparities exist in colon cancer treatment outcomes between African Americans and Caucasians. Studies indicate that African American individuals experience worse outcomes (decreased tolerance to therapy) when compared to Caucasians. Specifically, one study by McCollum et al. showed that hematological toxicities were significantly more common in African Americans compared with Caucasians. However, the overall incidence of other toxicities (diarrhea, nausea and vomiting and mucositis) were lower in African Americans compared to Caucasians.^76^ The ethnic disparity of hematological toxicities are attributed to lower levels of dihydropyrimidine dehydrogenase (DPD), the rate‐limiting enzyme of 5‐FU catabolism, in peripheral blood mononuclear cells.^77^ The prevalence of DPD deficiency has been shown to be 8.0% in African Americans, contrasted to only 2.8% among Caucasians. Currently, more than 30 SNPs and deletions within *DYPD* (gene encoding DPD) have been noted, while only a few cause enzymatic alterations.^78^ A large multicenteric prospective clinical trial on 683 patients treated with 5‐FU showed that one allele, DPYD*2A, can be linked to increased toxicity.^79^

Similar to DPD, thymidylate synthase (TS) enzyme level fluctuations based on ethnicity have been extensively studied.^80^, ^81^ Genetic changes modulating TS levels correlate with 5‐FU toxicity.^82^ Tandem repeats in the *TYMS* (gene for TS) enhancer region^83^ positively correlate with TS expression. For example, three copies of tandem repeat (TSER*3) result in approximately 2.6 times greater TS expression compared to two copies (TSER*2).^84^ Furthermore, individuals who were homozygous for double repeats displayed more severe side effects of 5‐FU. For instance, 67% of Chinese individuals express the TSER*3‐TSER*3 genotype when compared to only 30% to 40% of Caucasians, indicating a drastic difference in 5‐FU tolerability among the two populations.^80^ The population frequency of the TSER*3 allele in Caucasians (54%) is similar to that of African Americans (52%); however, two rare alleles (TSER*4 and TSER*9) are found at a higher frequency in African Americans (2%) than in Caucasians (0%).^81^ These studies indicate that ethnic differences play a significant role in the metabolism and toxicities of several antimetabolites. Genotyping prior to the administration of these chemotherapeutics in select populations may help prevent and predict toxicities. Furthermore, genotyping of set genes involved in drug metabolism could individualize treatment for patients. Specifics on antimetabolites are listed in Table 3.

**TABLE 3:** Pharmacokinetics, pharmacodynamics, and ethnic differences in the treatment outcomes with antimetabolite chemotherapy

Drug	Route, dosage form and dose (human)	Pharmacokinetics (PK)^a^	Pharmacodynamics (PD)^a^	Ethnic variability of PK/PD
Azacitidine	Intravenous Injection; 75 mg/m^2^	Cmax: 750 ng/mL (SC) Tmax: SC: 0.5 hr PO: 1 hr F SC: ~89% relative to IV PO: ~11% relative to SC Protein bound: ~6–12% (PO) Blood‐to‐plasma ratio: ~0.3 (PO) Vd: IV: 76 L, PO: 881 L M: hydrolysis and deamination via cytidine deaminase CL: 167 L/h (SC) t^1/2^ PO: ~0.5 h; SC or IV: 4 h E: PO: <2% unchanged (urine) IV: 85% (urine); <1% (feces) SC: 50% (urine)	Pyrimidine analog of cytidine that inhibits DNA/RNA methyltransferases Azacitidine is incorporated into RNA and DNA following cellular uptake and enzymatic biotransformation to nucleotide triphosphates Hypomethylation of DNA and direct cytotoxic effect on abnormal hematopoietic cells in bone marrow	Azacitidine was safe and effective in Chinese patients with myelodysplastic syndromes. Cmax concentrations were higher in Chinese patients when compared to North American patients.^85^
5‐fluorouracil	Intravenous Injection; 200–600 mg/m^2^	t^1/2^: 16 min Onset: 2–7 d Duration: 24 h M: liver (urea, fluorouracil, dihydrofluorouracil) E: urine	Prevents DNA synthesis in S phase by suppressing of thymidylate synthetase	Hematologic toxicities are more common in African Americans than in Caucasians; Adverse effects: Diarrhea, mucositis, and nausea/vomiting are more common in Caucasians than in African Americans.^77^	
6‐mercapto‐purine	Oral Tablets; 2.5 mg/kg	F: 5–37% Tmax: 2 h Protein Bound: 19% Vd: 0.56–0.9 L/kg M: GI mucosa, liver (6‐thiouric acid) Elimination t^1/2^: 21 minutes (children), 47 min (adult) CL: 11 mL/min/kg E: urine Dialyzable: no	Analog of naturally occurring purines, hypoxanthine and guanine Purine antagonist, antineoplastic	Adherence rates were significantly lower in African Americans (87%) and Asian Americans (90%), as compared with non‐Hispanic Caucasians (95%). Adherence at <90% was associated with a 3.9‐fold increased risk of relapse in a multiracial cohort of children.^86^	
Capecitabine	Oral Tablets; 2500 mg/m^2^	Oral F: 40% Tmax: 0.5 h (0.5–1.5 h range) Cmax: 22–29 ng/mL Protein bound: 20% Vd: 480–490 L M: Liver Elimination t^1/2^: 1 day Renal CL: 22.2 L/h E: Urine (28.5%)	The folate cofactor, N5‐10‐methylenetetrahydrofolate, and 5‐fluoro‐2′‐deoxyuridine monophosphate bind to thymidylate synthase to form a covalently bound ternary complex; this binding prevents the formation of thymidylate from 2′‐deoxyuridylate; since thymidylate is a prerequisite for the production of thymidine triphosphate, which is necessary for DNA synthesis, so that a deficiency of this compound can inhibit cell division	African Americans had lower rates of diarrhea compared to non‐Hispanic Caucasians .^87^	
Cladribine	Intravenous Injection; 1 mg/mL	t^1/2^: 5.4 h Protein bound: 20% Vd: 4.5 L/kg CL: 978 mL/h/kg E: urine Oral F: 40% Tmax: 0.5 h Cmax: 22–29 ng/mL Protein bound: 20% Vd: 480–490 L M: Liver Elimination t^1/2^: 1 day Renal CL: 22.2 L/h E: Urine (28.5%)	Purine analog, impairs DNA repair; cladribine is a prodrug; in the treatment of hairy cell leukemia, the active form incorporates into DNA, resulting in breakage of DNA strand and shutdown of DNA synthesis and repair, which in turn inhibits cell replication A prodrug containing the active component Cd‐ATP, cladribine is considered to have cytotoxic effects on B and T cells through impairment of DNA synthesis, leading to lymphocyte depletion in multiple sclerosis.	None reported	
Clofarabine	Intravenous Injection; 1 mg/mL	t^1/2^: 5.2 h Protein Bound: 47% Vd: 172 L/sq.meter M: intracellular CL: 28.8 L/h/sq. meter E: urine 49–60%	Purine nucleoside analog, suppresses ribonucleotide reductase & DNA polymerases Stimulate mitochondrial‐apoptosis	None reported	
Cytarabine	Intravenous, subcutaneous Injection; 5–75 mg/m^2^	Tmax: 20–60 min Peak CSF time (intrathecal): 60 min Peak CSF conc. (intrathecal): 30–50 mcg/mL Protein bound: 13% M: liver (major), kidneys (minor) Elimination t^1/2^: 1–3 h t^1/2^ (CSF after IT): 5.9–82.4 h CSF clearance rate: 0.24 mL/min E: urine	Metabolite cytarabine‐5′‐triphosphate suppresses DNA polymerase during S phase	African Americans were more susceptible to the growth‐inhibitory effects of cytarabine compared to Caucasians.^88^	
Decitabine	Intravenous Injection; 15 mg/m^2^	Protein Bound: <1% Vd: 63–89 L/m^2^ t^1/2^: 30–35 min	Suppresses DNA methyltransferase, resulting in hypomethylation of DNA & cellular differentiation or apoptosis	Myeloid leukemia treatment outcome of Asian patients is better versus Caucasian patients suggesting ethnic difference of efficacy of decitabine treatment.^89^	
Fludarabine	Intravenous Injection; 25 mg/m^2^	Peak plasma time: 2 h Vd: 96–98 L/sq. meter Protein bound: 19–29% Metabolites: 2‐fluoro‐ara‐ATP CL: 8.9 L/h/sq.meter E: urine	Fluorinated purine analog, suppresses DNA polymerase alpha	None reported	
Gemcitabine	Intravenous Injection; 1000 mg/m^2^	Vd: 50 L/m^2^ (following infusions lasting <70 minutes); 370 L/m^2^ (long infusions) M: Liver E: Urine (92–98%) t^1/2^: 1.7–19.4 h CL, Male: 92.2 L/h/m^2^ (29 years); 75.7 L/h/m^2^ (45 years); 55.1 L/h/m^2^ (65 years); 40.7 L/h/m^2^ (79 years) CL, Female: 69.4 L/h/m^2^ (29 years); 57 L/h/m^2^ (45 years); 41.5 L/h/m^2^ (65 years); 30.7 L/h/m^2^ (79 years)	Gemcitabine diphosphate reduces the quantities of deoxynucleotides, particularly dCTP, via inhibiting ribonucleotide reductase. Gemcitabine triphosphate and dCTP compete to be incorporated into DNA; the action of the diphosphate, which lowers the intracellular concentration of dCTP, increases the incorporation of gemcitabine triphosphate into DNA (self‐potentiation). One more nucleotide is only added to the expanding DNA strands after the gemcitabine nucleotide has been absorbed into the DNA, which finally causes the start of apoptotic cell death.	The prevalence of several genotypes in the pharmacology of gemcitabine varies between Caucasians and Asians. CDA + 435, and SLC28A1 + 1561 are worthy of further investigation as potential indicators of patient outcome after gemcitabine treatment in lung cancer.^90^	
Hydroxyurea	Oral Capsule or tablet; 15 mg/kg	Cmax: 1–4 h Vd: 0.5 L/kg Protein binding: 75–80% M: Liver and GI tract (60%) Elimination t^1/2^: 2–4 h E: Urine	Without affecting the production of ribonucleic acid or protein, hydroxyurea acts as a ribonucleotide reductase inhibitor to rapidly suppress the synthesis of DNA.	None reported	
Nelarabine	Intravenous Injection; 1500 mg/m^2^	Peak Plasma (adult dose): 2–8 mcg/mL Elimination t^1/2^ (adult dose): nelarabine 30 min; ara‐G 3 h M: Hydrolysis and demethylation E: partially in urine	First metabolized to the nucleoside analogous, ara‐G, and then to the nucleotide analogous, ara‐GTP, that binds to DNA and prevents DNA replication as well as causes cell death.	None reported	
Pemetrexed	Intravenous Injection; 500 mg/m^2^	Vd (ss): 16.1 L Protein bound: 81% M: Polyglutamate forms CL: 91.8 mL/min t^1/2^: 3.5 h E: Urine (70–90%)	Antifolate anticancer drug interferes with metabolic pathways that require folate and are crucial for cell growth.	African Americans ethnicity was not observed to be a major predictor of disease control following pemetrexed second‐line therapy in lung cancer compared to Caucasians.^91^	

Abbreviations: A, absorption; CL, clearance; Cmax, peak plasma or serum concentration; D, distribution; E, excretion; F, bioavailability; M, metabolism; t½, half‐life; Tmax, time to maximum concentration; Vd, volume of distribution.

^a^ PK/PD data of various drugs was obtained from ref. [65, 66, 67, 68].

## 7 ANTITUMOR ANTIBIOTICS

Antitumor antibiotics, also known as antineoplastic or anti‐cancer antibiotics, are developed from the products synthesized by Streptomyces bacteria. This class of medication works by halting cellular division through the specific inhibition of topoisomerase II, an enzyme responsible for cutting DNA to relieve any tangles and supercoils. These agents disrupt DNA and RNA primarily by binding to DNA via complexation.^92^ Antitumor antibiotics are often delivered intravenously; however, bleomycin can also be injected intramuscularly.

One investigation revealed that African Americans receiving anthracycline (doxorubicin, daunomycin, and mitoxantrone) treatment were at an elevated risk of cardiotoxicity, regardless of dose administered. The study revealed African Americans were at a 1.7‐fold higher relative risk of cardiotoxicity which included congestive heart failure, a reduction in observable cardiac function, or cardiac‐related death.^93^ A similar elevated risk of cardiotoxicity among African Americans who received antitumor antibiotics was further supported in another study, patients were given repeated doses of doxorubicin, with the final echocardiographic examination occurring after a median treatment period of 1.3 years. The depressed ejection fraction and/or heart failure were seen in 72% of the patients in this study.^94^ A different study revealed that African Americans are less likely than Caucasians to obtain the recommended number of cycles of therapy. African American patients were also more likely to terminate therapy earlier which resulted in reduced survival.^95^ Several SNPs that affect the pharmacokinetics of antitumor antibiotics have been found in two recent studies of Asian patients, however, the mechanism by which African Americans may be more susceptible to cardiotoxicity is unknown. The probable explanation is linked to the several polymorphisms in the *CBR1* (carbonyl reductase 1) and *CBR3* (carbonyl reductase 3) genes. These enzymes play a crucial role in conversion of doxorubicin to doxorubicinol, an active moiety that possesses the antineoplastic activity. However, doxorubicinol induces cardiotoxicity by removing iron from the catalytic Fe‐S cluster of the cytoplasmic aconitase (also known as iron regulatory protein‐ 1) which results in a non‐functional protein.^96^, ^97^, ^98^ Furthermore, a reduced expression of the IRP‐1 (iron regulatory protein) gene causes an increase in free iron levels and generation of reactive oxygen species.

Changes in CBR activity due to polymorphisms within *CBR* genes can have increased risk of anthracycline‐related cardiotoxicity. A specific polymorphism *CBR3* V244M has a 2.6‐fold higher rate of doxorubicinol synthesis and indicates that polymorphic CBRs can have a major impact on the pharmacodynamics of anthracyclines.^99^ Interestingly, there is no significant influence of *CBR3* polymorphisms on the pharmacokinetics of doxorubicin in Asian cancer patients. Further studies are needed to evaluate the role of CBR polymorphisms effect on treatment efficacy.

A comprehensive study involving transcriptomics, epigenomes, and SNPs of drug metabolism between Caucasians, African American, and Asians showed several changes in gene expression. Mutations of the *TP53, ATM, and CDK12 genes* showed significant resistance to chemotherapy, more specifically, TP53 mutation showed significant resistance to paclitaxel, 5‐Fluorouracil, doxorubicin, and gemcitabine. Furthermore, drug metabolism core genes (*CYP1A1, CYP2B6, CYP3A4, UGT1A8, UGT2B11, UGT2B17, UGT2B7, GAS5, XIST, SNHG6*) play a critically important role in chemotherapy resistance of prostate cancer. These genes also showed significant variation based on ethnicity, including *CYP1A1, CYP3A4, UGT1A8, UGT2B11, UGT2B17, GAS5, XIST, SNHG6* mutations were more prevalent in Caucasian individuals in comparison to Asian individuals; *UGT1A8, UGT2B17, UGT2B7, GAS5, XIST, SNHG6* were more significant in Black individuals in comparison to Asian individuals; and *CYP2B6, UGT1A8, UGT2B11, CYP3A4* were more significant in Caucasian individuals in comparison to Black individuals.^100^ Based on these findings, differences in drug response and treatment outcomes relate to interindividual drug metabolism which is influenced by ethnicity. Individualized treatment strategies may improve outcomes when taking these variations into consideration. Details regarding antitumor antibiotics medications are displayed in Table 4 below.

**TABLE 4:** Pharmacokinetics, pharmacodynamics, and ethnic differences in the treatment outcomes with antitumor antibiotics. PK/PD data of various drugs was obtained from ref. [65‐68].

Drug	Route, Dosage form and Dose [human]	Pharmacokinetics (PK)	Pharmacodynamics (PD)	Ethnic variability of PK/PD
Daunorubicin	Intravenous Injection; 45 mg/m^2^ Liposomal Formulation: 50 mg per vial (2 mg/mL)	Protein Bound: 50–60% t^1/2^: 14–20 h (parent drug), 24–48 h (daunorubicinol) Vd: 20–39.2 L/kg M: Hepatic, as daunorubicinol CL: 17.3 mL/min E: Feces (40%) & urine (25%)	Anthracycline: integrates across DNA base pairs, affects the topoisomerase II function and subsequently suppresses DNA and RNA replication.	Caucasians were more susceptible to cytotoxicity induced by daunorubicin in hematopoietic malignancies compared to African Americans.^101^, ^102^
Doxorubicin	Intravenous, intravesical Injection; 60–75 mg/m^2^ Liposomal formulation: 20 mg/m^2^	Protein Bound: 75% Vd: 20–30 L/kg (700–1214 L/m^2^) M: Predominantly liver as doxorubicinol Elimination t^1/2^: 1–3 h CL: 8–20 mL/min/kg E: Feces (40–50%); urine (5–12%)	Anthracycline: intercalates between DNA base pairs, impairs topoisomerase II function and inhibits replication & transcription	Increased cardiotoxicity is seen in African American patients with the treatment of Doxorubicin in non‐Hodgkin's lymphomas.^93^, ^94^
Epirubicin	Intravenous Injection; 100 mg/m^2^	t^1/2^: 31–35 h Protein Bound: 77% Vd: 21–27 L/kg M: Hepatic CL: 65–69 L/h E: Feces (34–35%) & urine (20–27%)	Anthracycline; integrates across DNA base pairs and prompts topoisomerase II cleavage, leading to cytocidal action. Suppresses DNA helicase and produces cytotoxic free radicals.	None reported
Bleomycin	Parenteral Injection; 0.25 unit/kg	t^1/2^: 2 h Protein Bound: 1% Vd: 17 L/m^2^ M: Bleomycin‐iron complex CL: 35 mL/min E: Urine (50–70%)	Glycopeptide antibiotic; hinders DNA, RNA, protein synthesis in G2, M phases	None reported
Dactinomycin	Intravenous Injection; 45 mcg/kg	Protein bound: Low M: minimal E: 30% in urine and feces	Actinomycin antibiotic; intercalates into DNA base pairs preventing RNA, DNA, and protein synthesis	None reported
Mitomycin‐C	Intravenous Injection; 20 mg/m^2^	t^1/2^: 48 min Vd: 16–56 L/m^2^ M: Liver CL: 201–810 mL/min/m^2^ E: Urine (10%)	Crosslinks DNA, preventing replication and transcription	In a multi‐ethnic Asian community, individuals with primary open angle glaucoma and primary angle closure glaucoma who had phacotrabeculectomy with mitomycin‐C often experienced hypotony, hyphema, and shallow anterior chamber.^103^
Mitoxantrone	Intravenous Injection; 12–14 mg/m^2^	t^1/2^: 5.8 day Protein Bound: 95% M: Hepatic Vd: >1000 L/m^2^; 14 L/kg E: Feces (25%); urine (11%)	Anthracenedione, a DNA‐reactive substance has non‐specific cytocidal action during the cell cycle that integrates into DNA to cause cross‐links and strand breakage, preventing the production of DNA and RNA.	African American patients are potentially more vulnerable to intercalating chemicals in breast cancer compared to Caucasian patients.^104^
Idarubicin	Intravenous Injection; 12 mg/m^2^	t^1/2^: 14–35 h (PO); 12–27 h (IV) F: 30% Protein Bound: 94–97% Vd: 64 L/kg Tmax: 1–5 h M: Liver as Idarubicinol CL: 122.8 L/h E: Urine (5–13%)	Anthracycline; intercalates between DNA base pairs, inhibits topoisomerase II, which in turn inhibits DNA and RNA synthesis	The Q141K polymorphism of the ABCG2 protein is related to poor outcome in adults with acute myeloid leukemia treated with idarubicin‐based chemotherapy, and Q141K‐ABCG2 is the most common variant among Caucasians. Similar to patients treated with Idarubicin who overexpress the wild‐type ABCG2 protein, Q141K is linked to poor outcomes.^105^ For Korean children with relapsed acute lymphoblastic leukemia, a weekly idarubicin dose of 10 mg/m^2^ in induction combination chemotherapy is results in lower treatment‐related mortality compared to the12.5 mg/m^2^ dose.^8^
Valrubicin	Intravesical Placement (into the bladder); 800 mg/week	M: as N‐trifluoroacetyl adriamycin, N‐trifluoroacetyl adriamycinol Excretion: Urine	Semi‐synthetic analog of doxorubicin which rapidly permeates cells but fails to seem to intercalate DNA; inhibits DNA topoisomerase II, which in turn suppresses DNA synthesis; produces significant chromosomal damage and arrests cells in the G2 phase of the cell cycle.	None reported

Abbreviations: A, absorption; CL, clearance; Cmax, peak plasma or serum concentration; D, distribution; E, excretion; F, bioavailability; M, metabolism; t½, half‐life; Tmax, time to maximum concentration; Vd, volume of distribution.

Historically antineoplastic agents are classified as (I) alkylating agents, (II) antimetabolites, (III) Antitumor Antibiotics, and (IV) miscellaneous. However, in recent years, the miscellaneous category has grown to encompass some of the essential agents, which are biological response modifiers, Histone Deacetylase inhibitors, Hormonal Agents, and Protein Kinase Inhibitors. Some of these chemotherapeutic PK/PD properties are not well studied within different ethnic populations.

### 7.1 Biologic response modifiers

Biologic response modifiers (BRMs) act as stimulatory or inhibitory immunomodulators that aid the body in fighting against cancer. These agents can occur naturally or be administered exogenously. Examples of BRMs include Aldesleukin (Interleukin‐2), Denileukin Diftitox (Interleukin‐2 with diphteria toxin), Pembroluzimab (PD‐1 inhibitor), and Interferon Gamma. As of today, there are no studies reported on PK and PD variations in different ethnicities using BRMs.

## 8 HISTONE DEACETYLASE INHIBITORS

Histone deacetylase (HDAC) inhibitors induce apoptosis via cell cycle arrest within cancer cells. HDACs remove acetyl groups present on histones to regulate DNA expression in the cell. When inhibited, gene expression becomes dysregulated creating issues down the line.^106^, ^107^ Each HDAC is a member of the SIR2 regulator family or the histone deacetylase family. Traditionally, HDACs are separated into distinct groups based on sequence similarity compared to yeast RPD3 or HDA1 protein. The sequences of the Class I proteins (HDAC1, HDAC2, HDAC3, and HDAC8) and the yeast Rpd3 protein are identical. The yeast Hda1 protein's sequence is comparable to that of the Class II proteins (HDAC4, HDAC5, HDAC6, HDAC7, HDAC9, and HDAC10). HDAC inhibitors can be manufactured or extensively purified from natural sources. Several HDAC inhibitors were recently given FDA approval for use as anti‐cancer medications. HDAC inhibitors include Belinostat, Panobinostat, Romidepsin, and Vorinostat..^108^

Two phase I studies studied the pharmacokinetics of Panobinostat specific to Japanese patients with advanced solid tumors. According to the study, all of the patients experienced thrombocytopenia and the severity depended on the dosage and their baseline platelet count. Within 8 days, the thrombocytopenia resolved. Plasma Panobinostat levels rose without clinically significant drug accumulation in a dose‐dependent manner. Six patients had stable illness for around 4 months, however, no one exhibited full or partial responses. In Japanese patients, oral Panobinostat administration at dosages up to 20 mg were well tolerated.^109^, ^110^

### 8.1 Hormonal agents

Hormones play a critical role in the development and progression of certain cancers, specifically those which rely on their influence for growth. Hormone therapy suppresses growth by blocking the anabolic effect of hormone stimulation. Hormone therapy is also known as endocrine therapy, hormonal therapy, or hormone treatment. Hormone therapy options include: (1) Antiandrogens: Abiraterone, Apalutamide, Bicalutamide, Cyproterone, Enzalutamide, Flutamide, Nilutamide. (2) Antiestrogens (including Aromatase Inhibitors): Anastrozole, Exemestane, Fulvestrant, Letrozole, Raloxifene, Tamoxifen, and Toremifene, (3) Gonadotropin Releasing Hormone Analogues: Degarelix, Goserelin, Histrelin, Leuprolide, Triptorelin, and (4) Peptide Hormones: Lanreotide, Octreotide, Pasireotide.

In a clinical study from Japan, 348 postmenopausal breast cancer patients received adjuvant anastrozole treatment for a median of 22 months. Anastrozole adverse effects included joint discomfort, bone mineral density (BMD) loss, and bone fracture. For both Japanese and Caucasian breast cancer patients treated with adjuvant anastrozole, the incidence of joint symptoms, overall clinical course, and risk factors were comparable. However, Japanese patients experienced a smaller reduction in BMD and a lower incidence of bone fractures after anastrozole treatment compared to Caucasian patients.^111^ Another study showed that shifting tamoxifen to anastrozole in postmenopausal Japanese breast cancer patients decreased the risk of disease recurrence.^112^ One study of lanreotide showed that the agent was well‐tolerated and efficacious in Korean patients with well‐differentiated gastroenteropancreatic‐neuroendocrine tumors.^113^

### 8.2 Protein kinase inhibitors

Protein kinases regulate the activity of cellular enzymes through phosphorylation. These proteins come in several varieties and interact with a wide range of cellular processes including cell growth, division, and signaling. Protein Kinase inhibitors currentl available are Abemaciclib, Acalabrutinib, Afatinib, Alectinib, Alpelisib, Axitinib, Binimetinib, Bortezomib, Bosutinib, Brigatinib, Cabozantinib, Carfilzomib, Ceritinib, Cobimetinib, Copanlisib, Crizotinib, Dabrafenib, Dacomitinib, Dasatinib, Duvelisib, Enasidenib, Encorafenib, Entrectinib, Erdafitinib, Erlotinib, Fedratinib, Futibatinib, Gefitinib, Gilteritinib, Glasdegib, Ibrutinib, and Idelalisib.^114^

One study analyzed Asian versus non‐Asian patients treated with Axitinib versus placebo. Based on ethnicity, treatment length (less than a year vs. more or equal than a year), dosage change, and severity of adverse events, disease‐free survival was examined. Analysis of the subgroups of Asian and non‐Asian patients found differences within medication exposure and adverse event occurrence. There were no variations in disease‐free survival or length of therapy related to ethnicity, however, decreased dosages of axitinib increased disease‐free survival. Compared to patients of non‐Asian descent, Asian patients displayed higher occurrences of nasopharyngitis, but showed less asthenia or fatigue. Among the subgroups of Asian patients, Japanese patients were more likely to have proteinuria, hypothyroidism, nasopharyngitis, and hypertension compared to Korean patients. Korean patients were more likely to have these conditions compared to Chinese patients.^115^

### 8.3 Tyrosine kinase inhibitors

Tyrosine kinase inhibitors (TKIs) are one of the newer forms of anti‐cancer agents available. Similar to PKIs, TKIs inhibit tyrosine kinase proteins which are involved in a variety of cellular functions involved with cell signaling, growth, and division. These agents have transformed cancer therapy by increasing survival benefit and quality of life while reducing toxicity profiles compared to conventional cytotoxic therapy. Despite having a specific mechanism of action, interindividual variation of the pharmacodynamic and pharmaceutics parameters exist between different ethnicities.^11^, ^116^, ^117^ TKIs administered at the same dose exhibit different systemic exposures, efficacy, and toxicity between individuals of different backgrounds.^118^ The differences observed from TKI administration can be explained by several intrinsic and extrinsic factors that alter the pharmacokinetic and pharmacodynamic ultimately altering treatment outcomes.^11^, ^119^

Individuals of European and East Asian descent appear to respond differently depending types of TKIs. Significant evidence shows that East Asian patients exhibit a greater response and improved survival outcomes to erlotinib, gefitinib, lapatinib, and regorafenib compared to individuals who are non‐East Asian.^120^, ^121^, ^122^, ^123^, ^124^, ^125^, ^126^, ^127^ As of now, no specific studies regarding inter‐ethnicity disparities of treatment response have been done for ruxolitinib and Osimertinib.^128^, ^129^, ^130^, ^131^ Additionally, certain TKIs display different tolerability profiles based on an individual's ethnicity. Some studies suggest that Asian patients experience higher rates of adverse events that are often more severe when compared to individuals from a European background. An exception of this is observed with ceritinib and crizotinib where Asian patients are less susceptible to adverse events.^128^, ^132^ Overall, those of East Asian descent demonstrate greater rates of toxicity‐related dose reductions, dose interruptions, and medication discontinuation compared to those of European descent.^121^, ^122^, ^133^, ^134^ Other than the studies mentioned above, there is little clinical comparison of PK parameters between different ethnicities to date.

### 8.4 Inter‐ethnicity differences in PK of tyrosine kinase inhibitors

The systemic exposure of orally administered TKIs varies by each drug's bioavailability. Bioavailability is influenced by cellular membrane transporters and metabolizing enzymes involved with the processing of TKIs.^9^, ^135^ Common efflux transporters such as P‐glycoprotein (P‐gp) and BCRP (Breast Cancer Resistant Protein) move most TKIs except cabozantinib, ibrutinib, regorafenib, ruxolitinib, and trametinib. With the exception of axitinib for OATP1B1 and OATP1B3, regorafenib for OATP1B1, nintedanib, and dasatinib for OCT1, in vitro studies indicate that the majority of TKIs cannot be regarded as substantial substrates for uptake transporters.^136^, ^137^ Some genetic factors that determine the variability of TKI response and toxicity include single nucleotide polymorphisms (SNPs), a premature stop codon, or altered splicing. For instance, the non‐synonymous SNPs 34G > A (Val12Met) and 421C > A (Gln141Lys) in the coding regions of *ABCG2* are associated with decreased BCRP expression and activity, reduced efflux and increased plasma and cellular exposure of several BCRP substrates.^137^, ^138^, ^139^, ^140^ Haplotypes (SNPs inherited together in a particular pattern on the same chromatid) can affect TKI pharmacokinetics. For instance, the *ABCB1* 3435 T > C/1236 T > C/2677 T > G/A TTT haplotype combination results in decreased P‐gp expression and has been associated with increased plasma exposure of P‐gp substrates such as TKIs.^141^

With a volume of distribution approximately between 100 and 1000 L and a terminal half‐life between 24 and 48 h, most TKIs demonstrate wide tissue distribution.^117^ Ultimately, membrane transporters play a critical role in the distribution of TKI. In order for TKIs to have effect, the agents must enter cancer cells and interact with intracellular molecular targets. Transporter‐mediated uptake into and efflux out of the target cells are crucial factors in TKI drug action and delivery.^9^

Cytochrome‐P450 (CYP) enzymes are primarily responsible for metabolizing TKI by phase I and UDP‐glucuronosyltransferases (UGTs) by phase II reactions.^142^ The enzyme CYP3A4 is principally responsible for metabolism of sunitinib and imatinib into the biologically active metabolites N‐desethyl (SU12662) and N‐demethylated piperizine and have similar potencies to their parent medications. Additionally, sorafenib and erlotinib are metabolized into their respective pharmacologically active metabolites, sorafenib N‐oxide and OSI‐420. Reduced transcription and reduced UGT1A1 enzyme activity is noted when there is presence of additional TA repeat in the TATA sequence of the UGT1A1 promotor (UGT1A1*28).^143^, ^144^ Similarly, polymorphisms in the nuclear receptors pregnane X receptor (PXR) and constitutive androstane receptor (CAR), which regulate the transcription of genes encoding drug metabolizing enzymes and transporters can be affected causing inter‐ethnic differences in metabolism.^9^, ^145^ More studies are needed to understand the diverse variability in metabolizing TKIs.

TKIs are largely excreted from the body by hepatic metabolism and P‐gp‐mediated biliary excretion, with less than 10% of total systemic clearance coming from unchanged drug elimination in urine.^142^ With the exception of afatinib, which is primarily excreted intact in feces, the majority of TKIs undergo substantial metabolic processing before biliary elimination.^146^ The biliary excretion of substrates of TKIs are facilitated by ABC transporters, which are expressed on the bile canaliculi of hepatocytes. They are additionally expressed on renal epithelial cells, where they facilitate the export of substances from the renal tubular cells' cytoplasm to the urine.^116^, ^136^, ^147^

### 8.5 Monoclonal antibodies

Another more recent therapeutic strategy against cancer is the use of monoclonal antibodies. Since the use of monoclonal antibodies is fairly new, several studies addressing the impact ethnicity has on the PK or PD parameters of anti‐cancer therapeutics have yet to be conducted.^11^ There are several types of monoclonal antibodies used to treat various types of cancer. The monoclonal antibodies approved and currently used include Alemtuzumab, Atezolizumab, Avelumab, Bevacizumab, Blinatumomab, Brentuximab, Cemiplimab, Cetuximab, Daratumumab, Dinutuximab, Dostarlimab, Durvalumab, Elotuzumab, Gemtuzumab, Inotuzumab Ozogamicin, Ipilimumab, Mogamulizumab, Moxetumomab Pasudotox, Necitumumab, Nivolumab, Ofatumumab, Olaratumab, Panitumumab, Pembrolizumab, Pertuzumab, Ramucirumab, Rituximab, Tositumomab, and Trastuzuma.^148^ These agents are further classified as bispecific antibodies (four) and Antibody‐drug Conjugates (ADC, one). Globally, two bispecific antibodies (cadonilimab and ozoralizumab) and another five monoclonal antibodies have been approved in China or Japan in 2022. Furthermore, another twenty‐four investigational monoclonal antibodies are currently undergoing scientific review by various regulatory organizations and are expected to be in the market in 2023. Thus, there is a strong acceptance for the use of monoclonal antibodies in the treatment of cancer. Monoclonal antibodies‐based immunotherapy is a pharmacologically recognized and encouraging therapy for hematological cancers and solid tumors. However, the pharmacogenetics of anti‐cancer monoclonal antibodies is sparse because of their novelty.

There are several polymorphisms in genes (FcgR3A gene‐rs396991 T/G, FcRn gene, BRAF gene, PTEN gene, VEGFA, RAS gene) or pathways (Ras/Raf/ERK, PI3K/AKT/mTOR) in patients that may enhance or decrease monoclonal antibody binding affinity and improve antibody‐dependent cell‐mediated cytotoxicity. As an example, human epidermal growth factor receptor (HER) 2‐positive breast cancer patients with the V/V genotype (FcgR3A gene‐rs396991) have a greater response rate to treatment with the anti‐HER2 monoclonal antibody, trastuzumab. Furthermore, this specific mutation improves the response rate and progression‐free survival of colorectal cancer and B‐cell lymphoma patients treated with cetuximab and rituximab. Compared to traditional chemotherapeutics, the metabolism of monoclonal antibodies does not involve the cytochrome P450 enzyme system. Based on the large molecular size of monoclonal antibodies, elimination occurs from uptake via receptor‐mediated endocytosis and pinocytosis which is followed by proteolytic catabolism.

In general, the efficacy of traditional chemotherapeutics vary primarily by polymorphisms observed in the genes responsible for transport and metabolism. However, inter‐individual differences in chemotherapeutic response of monoclonal antibody‐chemotherapeutics vary drastically. Based on the structural nature of the monoclonal antibodies (immunoglobulin G, protein) and the route of administration, their efficacy can be affected by alterations in genes responsible for antibody recognition and metabolism. Typically, the half‐life of monoclonal antibodies can range from days to months which depends on the structure. Monoclonal antibody main route of administrations include intravenous, subcutaneous, and intramuscular injections. Owing to the significantly elevated risk of infusion‐associated hypersensitivity/pharmacological reactions following IV route, SC, and IM are more preferable. Systemic absorption of monoclonal antibodies is gradual and relatively slow, and a maximum concentration occurs from 24 h to a week. The distribution of monoclonal antibodies occurs through the blood‐tissue hydrostatic gradient and through the sieving effect of the vascular epithelium. Moreover, the pharmacokinetic profile of monoclonal antibodies is mainly affected by age, BMI, gender, rate of respiration (respiratory rate), cardiovascular function (blood pressure), other coexisting pathologies (disease stage), and most importantly, the genetic profile. The potency, efficacy, and adverse drug reaction profile of monoclonal antibody chemotherapeutics varies due to ethnicity and genotypic alterations associated with the recognition, metabolism, and cancer cellular and molecular signaling pathways. Most of these studies were performed in America and Europe.

## 9 CHALLENGES IN DERIVING THE ETHNICITY/RACIAL DIFFERENCES DATA FOR CANCER DRUGS

**FIGURE 3:**
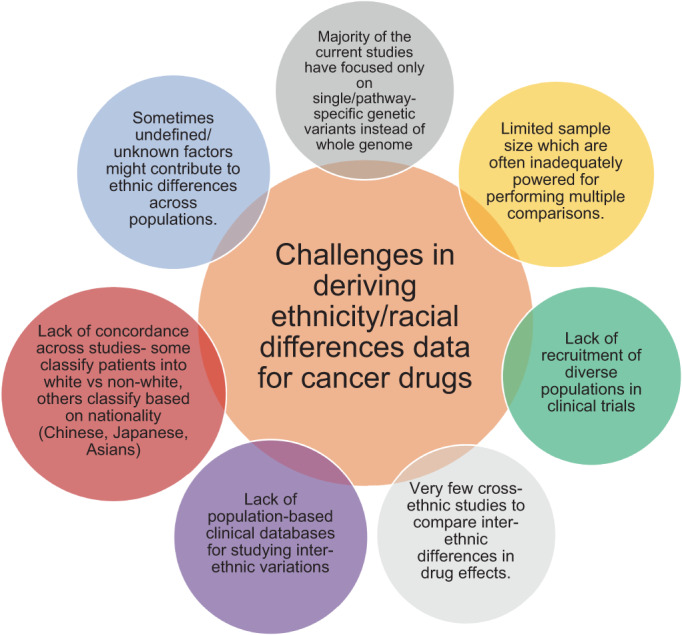
Various challenges in deriving the ethnicity/racial differences data for cancer drugs.

Several challenges arise when seeking data regarding the properties of chemotherapy from patients of different ethnicities. The challenges associated in deriving the ethnicity/racial differences data for cancer drugs are summarized in Figure 3.

1. Many studies compiled in this review have shown that there is considerable variation in PK/PD of chemotherapeutic drugs between populations of diverse ethnicities. For example, differences in genetic makeup, diet, lifestyle, and environmental factors can all affect how a drug is absorbed and metabolized in the body. This can lead to differences in drug efficacy and safety, as well as differences in the risk of adverse effects. Hence, it is important to consider the patient's ethnicity when prescribing medications. This may involve adjusting the dosage or choosing a different drug altogether. It is also important for researchers to study PK/PD in diverse populations to improve drug development and ensure that new drugs are safe and effective for everyone.
2. Race or ethnicity can influence the drug disposition, which can have an impact on both the drug's effectiveness and side effects. Both intrinsic and extrinsic factors can affect the drug disposition. Certain genetic variations are more common in certain ethnic groups and can affect the activity of enzymes responsible for metabolizing drugs. Additionally, differences in body weight, body surface area, and organ function can all affect the drug distribution and metabolism, which can impact the drug's effectiveness and toxicity. Besides, some ethnic groups may have dietary or cultural practices that affect their drug metabolism. Additionally, exposure to certain environmental toxins or pollutants may affect the drug metabolism. Overall, understanding the role of ethnicity in drug disposition and effectiveness is important for optimizing chemotherapy treatment and minimizing potential side effects.
3. Intrinsic factors include discrepancy within a person's physiology, genes, and pathology. As discussed earlier, several genetic polymorphisms exist at greater amounts in certain ethnicities. Genetic polymorphisms of enzymes, seen specifically in certain populations, also affect PK/PD data. Many of these SNPs influence the metabolism of these chemotherapeutics leading to overall changes in drug PK and PD parameters.
4. Extrinsic factors include culture, environment and diet, dosage formulation and regimens that have largely been disregarded in clinical trials. These factors might reveal variability in PK/PD data based on the absorption spectrum of different dosage formulations. PK/PD data variability can occur either by differences in first‐pass metabolism of the drug due to ethnicity or depend on the oral clearance due to the range of a drug's oral clearance. Similarly, changes in the dose interval impact PK/PD data. Similarly, dose formulation might trigger significantly different PK/PD responses in various ethnic groups due to their difference in susceptibility to several intrinsic and extrinsic factors. For example, decreased transdermal penetration of certain drugs have been reported in people with darker skin tones compared to people with paler skin populations,^149^ indicating the importance of considering route of administration into the clinical trials. In a similar study, Asians showed significantly higher AUC and Cmax than Caucasians^41^ after oral administration of single dose alprazolam. This might be due to higher CYP3A activity in Caucasians compared to Asians.^150^, ^151^ However, these differences were not significant when administered by IV route.^152^ Currently there are no chemotherapeutic drugs administered via skin, but there are many drugs undergoing preclinical and clinical studies.^153^ The topical delivery could be a better route for localized drug effects and to decrease the dose and dose frequency and to decrease the toxic side effects associated with oral or parenteral route of anti‐cancer drugs. Therefore, it is important to consider other relevant extrinsic factors (dosage forms, route of administration) that may influence the study results.
5. In addition to relevant intrinsic and extrinsic factors, another key factor for the success of all the studies is the appropriate sample size, that is, the number of subjects from each ethnic group should be sufficient enough to allow comparison of interethnic effects. In this regard, it is important to point out that majority of the clinical evidence obtained so far was only from three major ethnic groups: African Americans, Caucasians and Asians, thus ignoring other groups such as American Indians and Hispanics. As reported in 2020, the industry sponsored clinical trials of new biologics and drugs included only 8% of African Americans, 11% Hispanics or Latinos and 6%Asians.^154^ When the participants are homogenous, the results may be skewed leading to PK/PD data that is not generalizable. Therefore, it is important to consider large enough sample size for each of the ethnicities to draw meaningful conclusions on PK/PD data for different ethnicities. In order to advance equity in clinical trials for new drugs, there is a need to implement a combination of federal incentives and regulations such as providing grants and support for increasing enrolment capacity and infrastructure at trial sites and lowering the financial barriers by reimbursing the costs incurred by trial participants.^114^
6. Obtaining PK/PD data for different ethnicities is hindered by several challenges, including inadequate screening of subjects, small sample sizes, insufficient consideration of intrinsic and extrinsic factors, and the absence of population‐based clinical databases. These factors must be addressed to obtain accurate PK/PD comparisons for different racial/ethnic groups.^76^, ^155^, ^156^, ^157^, ^158^

## 10 CONCLUSION AND FUTURE PERSPECTIVE

Pharmacoethnicity impacts the treatment outcomes of patients undergoing various forms of anti‐cancer therapy. Primarily through polymorphisms within the genes responsible for metabolism, several PK and PD parameters can be impacted. These polymorphisms are observed at different frequencies within certain ethnic populations which lead to the wide range of outcomes observed after chemotherapy treatment. This paper discusses the clinical outcomes of different chemotherapeutics in regards to ethnicity. Ethnic diversity in chemotherapeutic drug response or toxicity is becoming increasingly recognized as an important healthcare burden globally. These differences can be attributed to both genetic and non‐genetic factors. Taking into account the significance of pharmacoethnicity will further the process of individualizing chemotherapy, which in turn will improve chemotherapeutic tolerance and efficacy. This review covers the current clinical literature for ethnicity specific differences in chemotherapeutic drug disposition (ADME), effectiveness, toxicity, and potential health outcomes from the treatment interventions. A multifaceted approach is necessary to address racial and ethnic disparities in chemotherapy outcomes. The clinical trials should cover diverse population groups/races/ethnicities. Furthermore, these disparities can be addressed by considering social determinants of health, developing personalized treatment plans, improving communication and cultural competence, and addressing implicit biases can help improve outcomes. These strategies can identify more effective treatments for specific racial and ethnic groups, ensure access to high‐quality cancer care for all patients, personalize treatment plans, improve communication, and address implicit biases among healthcare providers. All the concerned parties such as healthcare providers, researchers, and policymakers should work together and ensure that the patients receive appropriate care, drugs and dosing regiments to fight cancer.

## AUTHOR CONTRIBUTIONS

**Suhrud Pathak:** Data curation (equal); formal analysis (equal); methodology (equal); writing – original draft (equal); writing – review and editing (equal). **Kelsee Keyshu Zajac:** Data curation (equal); formal analysis (equal); methodology (equal); writing – original draft (equal); writing – review and editing (equal). **Manjusha Annaji:** Data curation (equal); formal analysis (equal); methodology (equal); writing – original draft (equal); writing – review and editing (equal). **Manoj Govindarajulu:** Writing – original draft (equal); writing – review and editing (equal). **Rishi Nadar:** Writing – original draft (supporting); writing – review and editing (supporting). **Dylan R Bowen:** Writing – original draft (supporting); writing – review and editing (supporting). **R. Jayachandra Babu:** Conceptualization (equal); data curation (equal); formal analysis (equal); funding acquisition (equal); resources (equal); writing – original draft (equal); writing – review and editing (equal). **Muralikrishnan Dhanasekaran:** Conceptualization (equal); data curation (equal); formal analysis (equal); funding acquisition (equal); resources (equal); writing – original draft (equal); writing – review and editing (equal).

### ETHICS STATEMENT

We searched for relevant literature and collected appropriate information for this review. The views expressed in the review are the opinions from one or more authors and all authors agree with the submitted content. All studies were appropriately cited and acknowledged.

## Data Availability

N/A
